# Low Surface Recombination in Hexagonal SiGe Alloy Nanowires: Implications for SiGe-Based Nanolasers

**DOI:** 10.1021/acsanm.3c05770

**Published:** 2024-01-12

**Authors:** Wilhelmus J. H. Willem-Jan Berghuis, Marvin A. J. van Tilburg, Wouter H. J. Peeters, Victor T. van Lange, Riccardo Farina, Elham M. T. Fadaly, Elsa C. M. Renirie, Roel J. Theeuwes, Marcel. A. Verheijen, Bart Macco, Erik P. A. M. Bakkers, Jos E. M. Haverkort, Wilhelmus M. M. Erwin Kessels

**Affiliations:** † Eindhoven University of Technology, Postbus 513, 5600 MB Eindhoven, The Netherlands; ‡ Eurofins Materials Science BV, High Tech Campus 11, 5656 AE Eindhoven, The Netherlands

**Keywords:** nanowires, hexagonal SiGe, surface passivation, SiGe, silicon photonics, surface recombination velocity, atomic layer deposition, direct bandgap

## Abstract

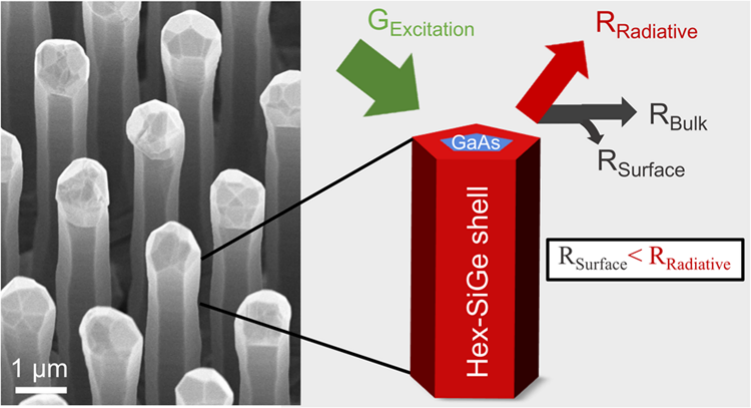

Monolithic integration of silicon-based electronics and photonics could open the door toward many opportunities including on-chip optical data communication and large-scale application of light-based sensing devices in healthcare and automotive; by some, it is considered the Holy Grail of silicon photonics. The monolithic integration is, however, severely hampered by the inability of Si to efficiently emit light. Recently, important progress has been made by the demonstration of efficient light emission from direct-bandgap hexagonal SiGe (hex-SiGe) alloy nanowires. For this promising material, realized by employing a nanowire structure, many challenges and open questions remain before a large-scale application can be realized. Considering that for other direct-bandgap materials like GaAs, surface recombination can be a true bottleneck, one of the open questions is the importance of surface recombination for the photoluminescence efficiency of this new material. In this work, temperature-dependent photoluminescence measurements were performed on both hex-Ge and hex-SiGe nanowires with and without surface passivation schemes that have been well documented and proven effective on cubic silicon and germanium to elucidate whether and to what extent the internal quantum efficiency (IQE) of the wires can be improved. Additionally, time-resolved photoluminescence (TRPL) measurements were performed on unpassivated hex-SiGe nanowires as a function of their diameter. The dependence of the surface recombination on the SiGe composition could, however, not be yet addressed given the sample-to-sample variations of the state-of-the-art hex-SiGe nanowires. With the aforementioned experiments, we demonstrate that at room temperature, under high excitation conditions (a few kW cm^–2^), the hex-(Si)Ge surface is most likely not a bottleneck for efficient radiative emission under relatively high excitation conditions. This is an important asset for future hex(Si)Ge optoelectronic devices, specifically for nanolasers.

## 1 Introduction

The continuous pursuit of technological progress has raised increasing interest in optical communication (e.g., optical interconnects^1−3^) and light processing devices (e.g., lab-on-a-chip^4^), which both require efficient light sources and detectors compatible with current silicon electronics. Unfortunately, one of silicon’s main drawbacks is its incapability to efficiently emit and absorb light. This has sparked interest in implementing other semiconductors on silicon substrate. Among the candidates are III–V materials, such as InP and GaAs. These materials have a direct bandgap, but they are rare and expensive^5^ and have a relatively large lattice mismatch with the Si substrate,^6^ which make them very difficult to integrate.

A new and very promising approach is based on germanium. This is a semiconductor that is chemically well matched to silicon and readily used in the semiconductor industry.^7^ Although the natural crystal structure of Ge (diamond cubic) does not exhibit a direct bandgap, it was predicted by Joannopoulos et al.^8^ that the hexagonal crystal structure does. More recent and much more accurate theoretical works have confirmed this prediction for hexagonal Ge^9^ and for hexagonal SiGe nanowires.^10^ Fadaly et al.^11^ finally showed with experimental evidence that the hexagonal crystal structure of Ge shows indeed efficient direct-bandgap emission. The use of GaAs/Ge core/shell nanowires (NWs) proved key to realizing hexagonal germanium (hex-Ge). Besides this, Fadaly et al.^11^ demonstrated that the direct bandgap is tunable from 0.3 to 0.7 eV by alloying the hex-Ge with 0–35% Si to create hexagonal silicon germanium (hex-SiGe).^11^ This makes hex-SiGe alloys a very promising candidate as silicon-compatible light emitters and absorbers, such as LEDs, lasers, and photodetectors compatible with Si-photonics circuitry. The scientific impact of hexagonal polytypes of Si and Ge has also been discussed in a recent review article.^12^

Optical characterization of the hexagonal GaAs/SiGe core/shell nanowires by Fadaly et al.^11^ revealed that the photoluminescence (PL) efficiency is significantly lower at room temperature (∼300 K) than at a few Kelvin (∼6 K). The latter suggests the presence of nonradiative recombination pathways. For nanostructures such as nanowires, surface-to-volume ratios are relatively large and surface defects can consequently play a much more pronounced role than in planar structures. Surface recombination via defects at the surface of the hex(Si)Ge crystals is therefore one of the primary suspects.

In this work, it is investigated whether and to what extent surface recombination is limiting the PL of hex-Ge and hex-SiGe alloys with the objective to clarify how the radiative emission of hex-SiGe at room temperature can be enhanced. It is however beyond the scope of this paper to investigate the detailed compositional dependence of the surface recombination mechanism since sample-to-sample variations of these state-of-the-art nanowires are presently too large to clearly observe compositional trends. For this purpose, temperature-dependent photoluminescence measurements were performed on nanowires with and without effectively proven surface passivation schemes. Case studies were performed for ultrathin (<25 nm) passivation layers and stacks that were earlier successfully studied on planar cub-Ge and cub-Si substrates: aluminum oxide (Al_2_O_3_),^13−15^ a stack of amorphous silicon and aluminum oxide (a-Si:H/Al_2_O_3_),^16^ and a stack of phosphorus oxide and aluminum oxide (PO_*x*_/Al_2_O_3_).^17−19^ These passivation schemes were selected as they have demonstrated very good passivation of cub-Ge and cub-Si surfaces with effective surface recombination velocities (*S*_eff_) between *S*_eff_ ≈ 300 cm/s and *S*_eff_ ≈ 2 cm s^–1^,^13−19^ which is very low compared to typical recombination velocities of, for example, InP surfaces (10^2^–10^4^ cm s^–1^)^20−24^ or GaAs surfaces (4 × 10^4^–10^7^ cm s^–1^).^25−28^ Besides this, these layers were selected because they are well documented and can be deposited by atomic layer deposition (ALD). This is a CMOS-compatible method that can provide excellent conformality on high-aspect-ratio structures such as nanowires. The latter makes these passivation layers preferred over, for example, an epitaxially grown Si passivation layer for which the required monolayer thickness control^29,30^ is highly challenging on 3D nanostructures such as these nanowires. The authors have also omitted higher-bandgap III–V semiconductors as a passivation layer since such materials are not compatible with the idea of using hex-SiGe for a Si-based laser. In addition to the investigation of these passivation schemes, time-resolved photoluminescence (TRPL) measurements were performed on unpassivated hex-SiGe nanowires as a function of their diameter. With these experiments, we demonstrate that at room temperature, under high excitation conditions (a few kW cm^–2^), no large improvements or degradations of the PL occur after applying the passivation schemes. Moreover, no increase in lifetime was found with an increasing nanowire diameter. These findings suggest that in this regime the surface of the hex-(Si)Ge is most likely not a bottleneck for efficient radiative emission, which is an important advantage for future hex-(Si)Ge optoelectronic devices such as nanolasers.

## 2 Experimental Details

### 2.1 Sample Characteristics

**Figure 1:**
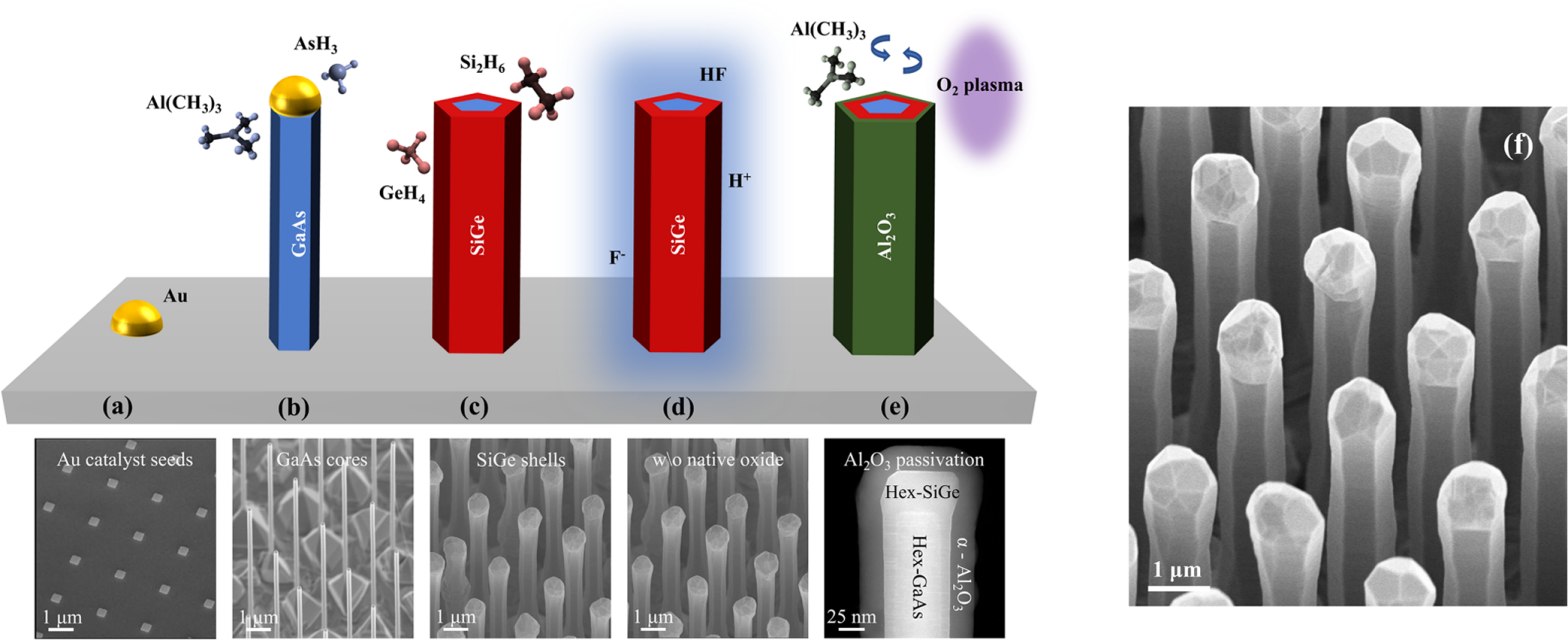
Schematic representation of the growth and passivation of GaAs/SiGe core/shell nanowires: (a) Gold particles on a GaAs substrate act as catalyst for vapor–liquid–solid (VLS) growth of wurtzite GaAs core nanowires. (b) Ga(CH_3_)_3_ and AsH_3_ are employed as precursor gases for the VLS growth of the GaAs nanowire cores. (b) Removal of the gold particles and growth of the SiGe shell by MOVPE using GeH_4_ and Si_2_H_6_ as precursor gases. (d) Removal of the native surface oxide by a wet chemical treatment (HF solution in this case). The abbreviation W.O. stands for “without”. (e) Surface passivation step. In this case by conformal growth of Al_2_O_3_ using an ALD process employing Al(CH_3_)_3_ as Al-precursor and O_2_ plasma as oxygen source. (f) Typical scanning electron microscopy (SEM) image of the nanowires studied in this work. This particular SEM image was taken of sample H6948 (see Table 1 for details). This figure is inspired by “Extended data Figure 1” from the work of Fadaly et al.^11^ The latter has been used with permission of Springer Nature, from Direct-bandgap emission from hexagonal Ge and SiGe alloys, Fadaly et al.; permission conveyed through Copyright Clearance Center, Inc.

The hexagonal GaAs/SiGe core/shell nanowires studied in this work were grown using an identical fabrication method as described by Fadaly et al.^11^ (Figure 1). The SiGe material grown in this way has degenerate *n*-type doping^11^ with a concentration that is estimated to be between 3 × 10^17^ and 3 × 10^18^ cm^–3^. For each experiment presented in the main text of this work, detailed information about nanowire characteristics can be found in Table 1. For the nanowires, several proven and well-documented passivation films were investigated, including a 22 nm thick film of Al_2_O_3_, a stack of 2 nm a-Si:H capped with 11 nm Al_2_O_3_ (a-Si:H/Al_2_O_3_), and a stack of 4 nm PO_*x*_ capped with 10 nm Al_2_O_3_. Information about these passivation schemes, including information about the surface treatment prior to deposition and the annealing treatment after deposition, is given in Table 1. Additional details can be found in the work of Berghuis et al.,^13^ Berghuis et al.,^16^ and Theeuwes et al.,^17^ respectively. The conformality of the passivation layers on the nanowires was examined using a JEOL ARM 200F Transmission Electron Microscope (TEM) operated at 200 kV and equipped with a 100 mm^2^ Centurio SDD energy-dispersive X-ray (EDX) spectroscopy detector.

**Table 1:** Overview of the hex-SiGe Nanowire Samples Used for Each Experiment in This Work^a^

experiment	sample name	nanowire composition	diameter (μm)	length (μm)	passivation scheme	surface treatment	*T*_PDA_ (°C)	*S*_eff_ (cm s^–1^) on cub-Ge	*S*_eff_ (cm s^–1^) on cub-Si
passivation	H05916	Si_0.23_Ge_0.77_	0.43 ± 0.07	1.6 ± 0.4	Al_2_O_3_ (22 nm)	1% HF_(aq)_	425	∼300^13^	<6^14,15^
passivation	H07771	Si_0.23_Ge_0.77_	2.08 ± 0.03	8.3 ± 0.3	a-Si:H/Al_2_O_3_ (2/11 nm)	20% HBr_(aq)_	325	∼2.7^16^	∼1.8^b^
passivation	H06950	Si_0.23_Ge_0.77_	0.617 ± 0.02	5.62 ± 0.02	PO_*x*_/Al_2_O_3_ (4/10 nm)	none^c^	250	∼8.9^17^	<6^18,19^
diameter series	H06950-2	Si_0.23_Ge_0.77_	0.617 ± 0.02	5.62 ± 0.02					
diameter series	H06948	Si_0.23_Ge_0.77_	1.0 ± 0.1	5.2 ± 0.9					
diameter series	H06988	Si_0.23_Ge_0.77_	1.2 ± 0.1	5.86 ± 0.07					
TEM	H05895	Si_0.2_Ge_0.8_	0.06 ± 0.005	1.7 ± 0.1					

a Both the nanowire characteristics and the passivation schemes applied to them are listed. The listed pre-deposition surface treatments and the post-deposition anneal temperatures (*T*_PDA_) were optimized for the surface passivation of each passivation scheme (see earlier work^13,16,17^). The last columns show the surface recombination velocity (*S*_eff_) as determined on planar cubic germanium and silicon wafers. Surface passivation of cubic SiGe has been investigated by several authors;^31−33^ however, surface recombination velocities of planar cubic SiGe are still very rarely reported in the literature and are therefore not given in the table.

b Note that for cub-Si and cub-Ge the optimum growth conditions for a-Si:H regarding surface passivation are slightly different.

c The native oxide is removed in situ by the PO_*x*_/Al_2_O_3_ deposition process through a self-cleaning effect.^17^

### 2.2 Optical Characterization

Information about (surface-related) nonradiative recombination of the nanowires was obtained via time-resolved PL and temperature-dependent PL. For the latter, the integrated PL (normalized at a low temperature, typically at a few Kelvins) was measured as a function of inverse temperature. In this way, the normalized PL represents the internal quantum efficiency or photoluminescence efficiency (η_PL_) of the nanowires. This quantity relates in a quantitative way to both radiative (*R*_r_) and nonradiative recombination rates (*R*_nr_):
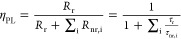
1with τ_r_ and τ_nr_ the lifetimes of the radiative and nonradiative processes, respectively. The thermal activation energy of the nonradiative recombination mechanisms can typically be described by the Arrhenius relation for thermal activation.^34,35^ The temperature dependence of the internal quantum efficiency has consequently the form of:^34−36^
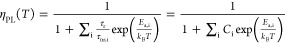
2with *E*_a,i_ the activation energy of the ith nonradiative recombination process and *C*_i_ the ratio of the radiative and nonradiative lifetime of the process, which is also known as the quenching rate. Both the radiative lifetime and C_*i*_ are expected to be independent of temperature.^37^ The quenching rates represent the strength of the nonradiative recombination and can be used to quantify potential reductions in nonradiative (surface) recombination as a consequence of surface passivation.

**Figure 2:**
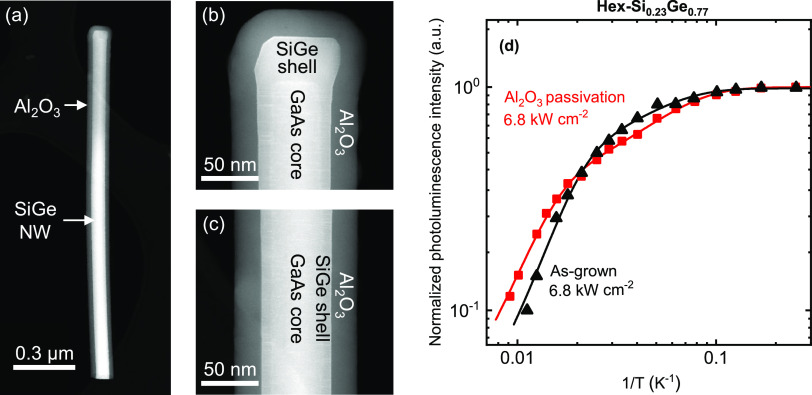
(a) High-angle annular dark-field scanning transmission electron microscopy (HAADF-STEM) image of a hexagonal SiGe nanowire (Si_0.2_Ge_0.8_, length ≈ 1.7 μm, diameter ≈ 60 nm, AR ≈ 27, pitch ≈ 2 μm, see Table 1) coated with approximately 22 nm PEALD Al_2_O_3_. Note that to enable this TEM image a thinner nanowire was used than for the PL measurements; see details in Table 1. (b) Close-up of the top part of the nanowire. (c) Close-up of the lowest part of the nanowire. (d) Arrhenius representation of the photoluminescence intensity as a function of inverse temperature for hex-Si_0.23_Ge_0.77_ nanowires without and with Al_2_O_3_ passivation film. The integrated PL intensities are obtained for an excitation density of 6.8 kW/cm^2^ and normalized to their respective intensity at 4 K. The data has been fitted with the Arrhenius equation (solid lines through the data, parameters listed in Section 2 in the Supporting Information).

**Figure 3:**
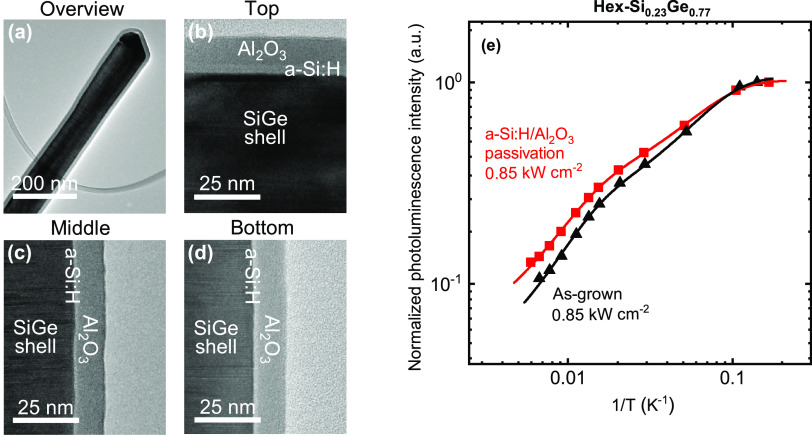
(a) Bright-field scanning transmission electron microscopy (BFTEM) image of the upper half of a hexagonal GaAs/SiGe core/shell nanowire (Si_0.2_Ge_0.8_, length ≈ 1.7 μm, diameter ≈ 60 nm, AR ≈ 27, pitch ≈ 2 μm, see Table 1) coated with the a-Si:H/Al_2_O_3_ stack. Note that to enable this TEM image, a thinner nanowire was used than for the PL measurements, see details in Table 1. (b) Close-up of the top part of the nanowire. The a-Si:H interlayer is approximately 3.0 ± 0.5 nm thick, while the Al_2_O_3_ capping is 10.5 ± 0.5 nm. (c) Close-up of the middle part of the nanowire (∼1 μm from the top). (d) Close-up of the lowest part of the nanowire (∼2 μm from the top). The a-Si:H interlayer measures 1.5 ± 0.5 nm and the Al_2_O_3_ capping 10.5 ± 0.5 nm. (e) Arrhenius representation of the photoluminescence intensity as a function of inverse temperature for hex-Si _0.23_Ge_0.77_ nanowires with and without a-Si:H/Al_2_O_3_ passivation stack. The integrated PL intensities are normalized to their respective intensity at 7 K. The data are fitted with the Arrhenius equation (solid lines through the data, parameters listed in Section 2 in the Supporting Information). An excitation density of 0.85 kW/cm^2^ is used.

For the temperature-dependent PL measurements, substrates with nanowire arrays (typical pitch of ∼2 μm) were mounted in a helium-flow cryostat. A 976 nm laser with a spot size of ∼40 μm was employed to excite the wires. The PL was spectrally resolved using a Fourier transform infrared (FTIR) spectroscopy setup containing a HgCdTe detector (used for the hex-Ge samples) and an extended InGaAs detector (used for the hex-SiGe samples). The signal contributions from the laser and any cub-(Si)Ge from the substrate were filtered out using appropriate long-pass filters. The integrated PL as reported in Figures 1, 2, and 3 was obtained without spectrally resolving the PL, but by modulating the excitation laser at 38 kHz and reading the PL response of the lock-in amplifier. The pumping power for an experiment was chosen to be much lower than required for observing stimulated emission^38^ so that surface recombination effects are more pronounced. On the other hand, the pumping power was chosen high enough to be able to observe a sufficient PL response from the sample(s) at room temperature or a temperature as close as possible to room temperature. The latter guarantees a sufficient PL response over the whole range of temperatures to be measured in the temperature-dependent measurements.

For the TRPL measurements, individual SiGe wires were mechanically placed onto a gold-coated silicon wafer. A SiO_2_ layer was deposited on top of the gold to prevent direct contact between the wires and the gold. The individual wires were excited with a femtosecond pulsed laser operating at a wavelength of 1030 nm with a pulse frequency of 40 MHz, a pulse width of 100 fs, and a spot size of ∼3 μm. A superconducting nanowire single-photon detector (SNSPD) from the brand Single Quantum was used to measure the PL signal as a function of time. The reflection from the laser was filtered out using a 1350 nm pass filter. The lifetime is extracted from the time-resolved PL measurements by fitting the PL decay as a function of time with a mono-exponential decay.

## 3 Results

### 3.1 Effect of Al_2_O_3_ Passivation

Figure 2a–c demonstrates that excellent conformality of ALD Al_2_O_3_ can be obtained on hex-SiGe nanowires. The figure shows a TEM image of a single hexagonal SiGe nanowire with an aspect ratio ∼27 after it has been subjected to 200 cycles of the ALD Al_2_O_3_ process. The wire, originating from a nanowire array with a pitch of ∼2 μm, shows along its complete length an equally thick Al_2_O_3_ film. As expected, the ALD process allows thus for good conformality on these nanostructures. The film measures about 22 nm, which is in line with the nominal growth per cycle (GPC) of this process (GPC ≈ 1.1 Å/cycle).^39^ Earlier work by Fadaly et al.^11^ (extended data Figure 6b,d) shows no signs of clustering or segregation of either Si or Ge in the hexagonal SiGe nanowires. Energy-dispersive X-ray spectroscopy (EDX) performed in this work on similar SiGe nanowires reveals that after deposition of ALD Al_2_O_3_, no clear signs of clustering or segregation appear (Section 1 in the Supporting Information).

The integrated photoluminescence of hex-Si_0.23_Ge_0.77_ nanowires with and without an Al_2_O_3_ passivation film is shown in Figure 2d. Typical spectra can be found in Section 3 in the Supporting Information. Figure 1d shows the integrated PL as a function of inverse temperature for SiGe NWs with and without an Al_2_O_3_ passivation film. Recall that the latter can suppress surface recombination velocities down to *S*_eff_ ≈ 300 cm/s on cub-Ge (Table 1). The PL intensities of the as-grown wires (black) and the passivated wires (red) were normalized at 4 K. The latter implies that the vertical axis also represents the internal quantum efficiency. As expected, the internal quantum efficiency decreases with increasing temperature due to the activation of nonradiative recombination mechanisms. For both the passivated and unpassivated wires, the quenching of the PL with temperature seems to be characterized by two knees. This implies the presence of two separate nonradiative recombination channels. Consequently, the data is fitted with the Arrhenius eq (eq 2, see Section 2) using two terms, yielding activation energies of *E*_a,1_ ≈ 3.2–3.8 meV and *E*_a,2_ ≈ 25–27 meV (details of fit parameters such as these activation energies can be found in Section 2 in the Supporting Information). The fits are represented by solid lines in Figure 2d. When the passivated and as-grown wires are compared, it becomes clear that the PL of the Al_2_O_3_ passivated wires shows less quenching with increasing temperature. This is also reflected by the slightly lower quenching rate for the passivated wires (*C*_2,pass_ ≈ 100 au vs *C*_2,unpass_ ≈ 183 au) obtained by the Arrhenius fit, and the 66% higher absolute PL intensity at the highest measurable temperature (90 K). If the nonradiative recombination can be (partially) attributed to the surface, then there is a slight reduction of the surface recombination.

### 3.2 Effect of a-Si:H/Al_2_O_3_ Passivation

Figure 3a–d shows TEM images of a single hexagonal SiGe nanowire after it has been subjected to 40 s Plasma-Enhanced Chemical Vapor Deposition (PECVD) a-Si:H and subsequently 100 cycles of plasma-enhanced ALD Al_2_O_3_. The properties of the nanowire are identical to those described in Figure 1a–c. The Al_2_O_3_ capping layer has an equal thickness of 10.5 ± 0.5 nm along the whole length of the nanowire, which indicates a very conformal coating. The PECVD a-Si:H interlayer is present along the whole nanowire, as well, which is important. When considering the film thickness, it is observed that the a-Si:H is substantially thicker on the top (3.0 ± 0.5 nm) than on the side walls (1.5 ± 0.5 nm). The lower thickness on the sides of the nanowire can be attributed to a lower flux of reactive plasma species during PECVD of a-Si:H.

The effect on the integrated PL of the a-Si:H/Al_2_O_3_ stacks, which can suppress recombination velocities on cub-Ge down to *S*_eff_ ≈ 3 cm/s (Table 1), is shown in Figure 3e. The integrated PL intensities of the as-grown hex-SiGe wires (black) and the passivated wires (red) have been normalized at 7 K (typical spectra are shown in Section 3 in the Supporting Information). The PL quenching with temperature in Figure 3e seems to be characterized by two knees for both the passivated and unpassivated wires, similar to that in Figure 2d. The Arrhenius fits of the data (solid lines) yield for the activation energies (*E*_a,1_, *E*_a,2_) of the as-grown and passivated wires similar values of 2.69 and 2.58 meV for *E*_a,1_ and 22 and 21 meV for *E*_a,2_. When comparing the passivated and as-grown wires, it becomes evident that they show very similar behavior. The PL intensity of the passivated wires decreases a little bit less with increasing temperature (*C*_2,pass_ ≈ 20 au vs *C*_2,unpass_ ≈ 30 au). This hints at a reduction in nonradiative recombination pathways, presumably related to the surface. The reduction is, however, slight. Also note that for even higher excitation densities, nonradiative recombination becomes relatively less important. The latter implies that for higher excitation densities, the differences between the quenching rates of the passivated and as-grown wires will be reduced to an even smaller difference. Another experiment performed with wires of pure hexagonal germanium also shows this experimentally (Section 3 in the Supporting Information).

### 3.3 Effect of PO_*x*_/Al_2_O_3_ Passivation

**Figure 4:**
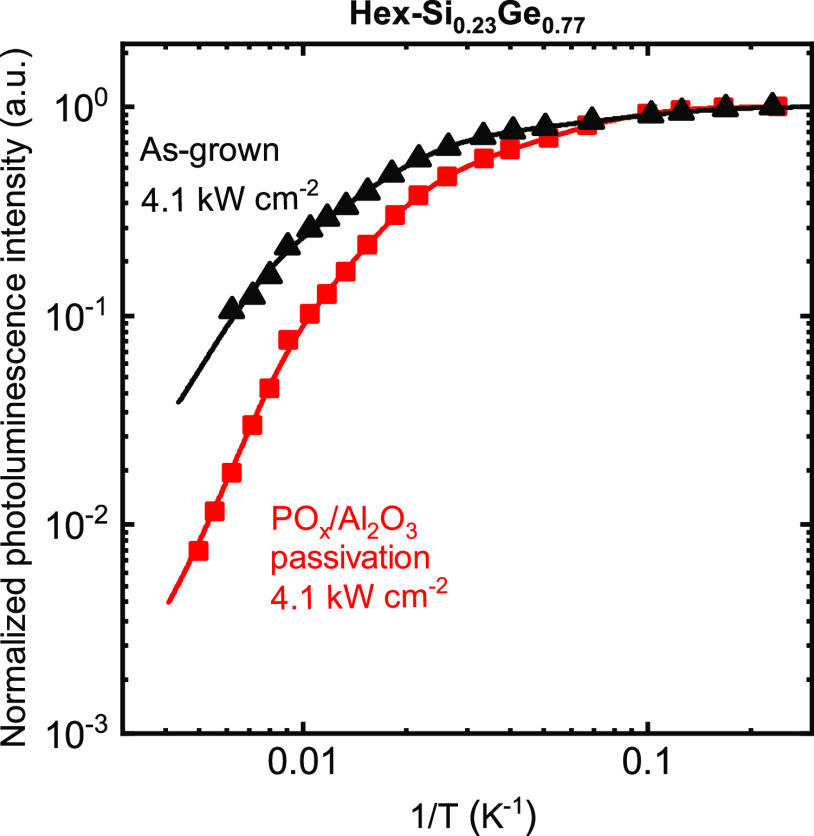
Arrhenius representation of the photoluminescence intensity as a function of inverse temperature for hex-Si_0.12_Ge_0.88_ nanowires without and with a PO_*x*_/Al_2_O_3_ passivation stack. The integrated PL intensities are obtained for an excitation density of 4.1 kW/cm^2^ and normalized to their respective intensity at 4 K. The data is fitted with the Arrhenius equation (solid lines through the data, parameters listed in Section 2 in the Supporting Information).

The conformality of the PO_*x*_/Al_2_O_3_ deposition process used in this work has already been demonstrated on nanowires in earlier work by Black et al.^20^ and Theeuwes et al.^40^ For this stack, we therefore focus only on the changes in photoluminescence as a consequence of applying this stack on the surface of hex-SiGe nanowires. Figure 4 shows the integrated PL intensity as a function of the inverse temperature for hex-Si_0.12_Ge_0.88_ nanowires without (black) and with a PO_*x*_/Al_2_O_3_ passivation stack (red). The PL intensities are normalized with respect to their intensities at 4 K. Note that the PO_*x*_/Al_2_O_3_ stack yields very low recombination at surfaces of cub-Ge (*S*_eff_ ≈ 9 cm/s (Table 1)). The temperature behavior of the integrated PL can be described by the Arrhenius equation using three terms, as observed before for this exact growth process of hex-SiGe nanowires with this composition.^11^ From Figure 4 it is, however, clear that the PL of the wires without the passivation stack decreases substantially less with increasing temperature. Additionally, the absolute integrated PL at 160 K (the highest measurable temperature) is 40% lower after applying the passivation film. These observations indicate no reduction in the level of surface recombination by the PO_*x*_/Al_2_O_3_ stack. Yet, there are some not fully understood observations for this case, which are discussed in Section 3.3 in the Supporting Information.

### 3.4 Screening of Additional Passivation Schemes

**Figure 5:**
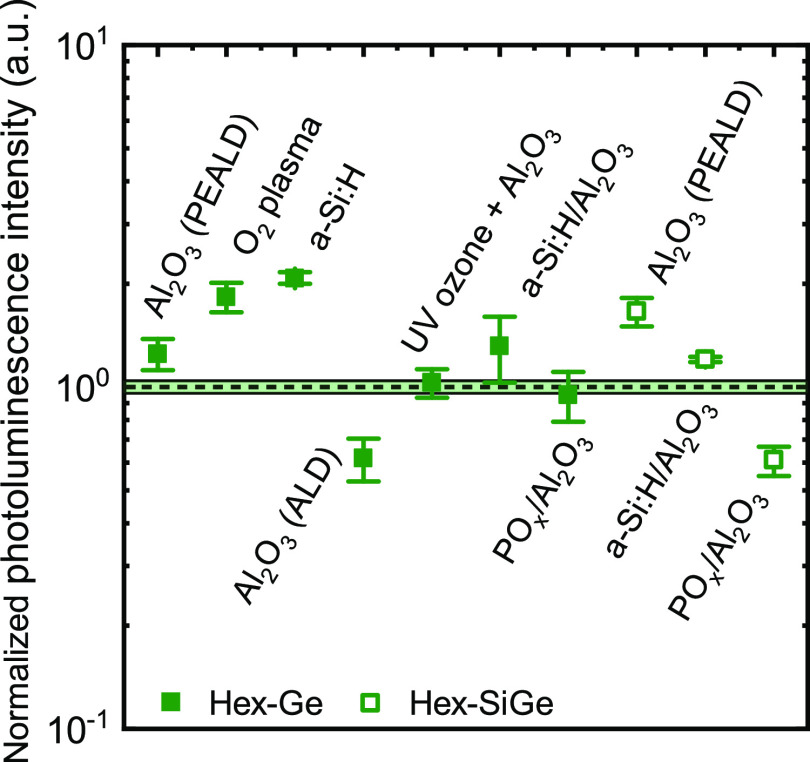
Overview of the integrated photoluminescence of various hex-Ge (closed symbols) and hex-SiGe (open symbols) nanowire ensembles passivated with various passivation schemes. The integrated PL is normalized with respect to the integrated photoluminescence of the same nanowires without a passivation scheme. The nanowires are measured at 293 K with the exception of the SiGe nanowires with the Al_2_O_3_, a-Si:H/Al_2_O_3_, and PO_*x*_/AlO_*x*_ passivation for which the highest measurable temperatures were 90, 149, and 160 K, respectively. Excitation densities used for the measurement vary slightly per sample but lie between 1 and 7 kW cm^–2^. The exact dimensions and composition of the nanowires and the measurement conditions associated with each data point are listed in Section 4 in the Supporting Information.

Besides the earlier discussed passivation schemes (Al_2_O_3_, a-Si:H/Al_2_O_3_, PO_*x*_/Al_2_O_3_), several variations on these passivation schemes have been screened as well. These were also all capable of realizing relatively low surface recombination velocities on cub-Ge and cub-Si. In Figure 5, the effect of all of these passivation schemes on the integrated photoluminescence of hex-Ge and hex-SiGe nanowires is presented. The integrated photoluminescence of the passivated wires is displayed with respect to the same nanowires without the passivation film (dashed line), i.e., a normalized PL intensity is displayed. The data is collected at room temperature (293 K) at relatively high excitation densities (≈1–7 kW cm^–2^). Details about the nanowires and the different passivation schemes are provided in Section 4 in the Supporting Information. Figure 5 shows that in most cases the passivation film slightly enhances the PL except for the thermal ALD Al_2_O_3_ film and the PO_*x*_/Al_2_O_3_ stack. Second and more generally, the presented passivation schemes show each a relatively mild impact on the integrated photoluminescence of the hex-Ge and hex-SiGe nanowires. To be more precise, the deviation from the as-grown nanowires (dashed line) is mostly within a factor of 2. The latter indicates a relatively small influence of the surface on the photoluminescence.

### 3.5 Lifetime vs Nanowire Diameter

**Figure 6:**
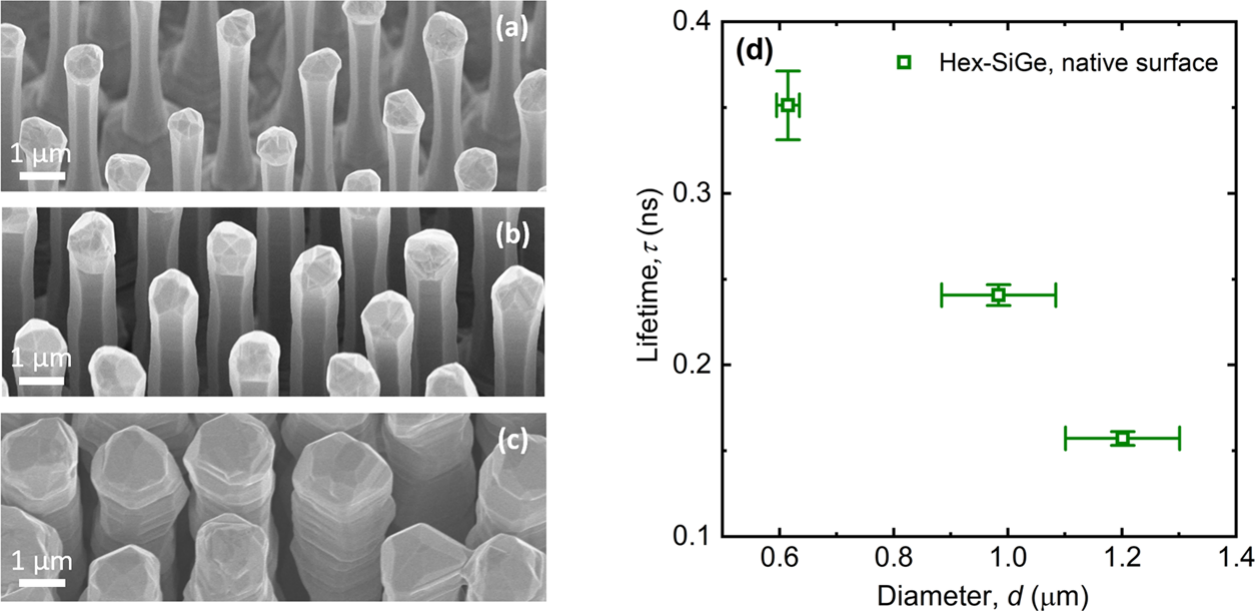
(a–c) SEM images of the samples H06950-2, H06948, and H06988. The ensembles feature nanowires with different thicknesses (diameters read 0.617 ± 0.02, 1.0 ± 0.1, and 1.2 ± 0.1, respectively). Individual nanowires were taken from these ensembles and studied in (d). More details about these samples can be found in Table 1. (d) Weighted average lifetime of individual Si_0.23_Ge_0.77_ nanowires as a function of their diameter at room temperature (≈300 K). The wires were individually excited with 1.0 mJ cm^–2^ pulses. For each data point, about 5–7 nanowires were averaged (raw TRPL data can be found in Section 5 in the Supporting Information). The standard deviation of the measured lifetimes was used to determine the error bars.

To further elucidate the importance of the surface, TRPL measurements were performed on individual unpassivated nanowires to determine the carrier recombination lifetime as a function of their diameter. The results of the experiments are presented in Figure 6. Figure 6a–c shows SEM images of the nanowire ensembles from which individual nanowires were taken and studied within this experiment (Section 2). In Figure 6d, the weighted average lifetime (τ) as a function of nanowire diameter at room temperature is displayed. For each data point, about 5–7 nanowires were averaged. The composition of SiGe nanowires is chosen to be Si_0.23_Ge_0.77_, which is approximately in the middle of the composition range that yields a direct bandgap (Si_0_Ge_1_–Si_0.35_Ge_0.65_). From Figure 6d, a clear correlation becomes visible between the diameter and lifetime: the thicker the nanowires, the lower the lifetime. This is exactly the opposite of what one expects if the lifetime is surface-limited. This data suggests therefore that at room temperature the native hex-SiGe surface is not the limiting factor but rather nonradiative recombination centers in the bulk. These bulk recombination centers may not be distributed homogeneously throughout the nanowire and may increase with diameter, leading to the diameter dependence of the lifetime as observed in Figure 6d. This was for example the case for the so-called I3 basal stacking fault,^41^ a structural defect in hex-SiGe that does however not act as a recombination center.^41^ Since the As concentration is rather homogeneous after the first 20 nm from the GaAs core,^11^ the diameter dependence of the lifetime seems unlikely to be related with relatively high and unintentional As content. Note finally that for temperatures substantially lower than room temperature, it was shown by Fadaly et al.,^11^ that recombination becomes purely radiative; i.e., nonradiative recombination is no longer dominant. According to their work, typical temperatures below which recombination becomes purely radiative are <40 K or even <200 K depending on the sample.^11^ In this work, the transition temperature for the wires presented in Figures 2, 3, and 4 seems to be around 50 K.

## 4 Discussion

When considering the nanowires that were studied in more detail (Figures 2–4), we observe some improvements by the Al_2_O_3_ and a-Si:H/Al_2_O_3_ passivation schemes. The effect of the PO_*x*_/Al_2_O_3_ passivation scheme remains somewhat less clear. Despite some influence of the passivation schemes, the quenching of the PL from 4 K to room temperature remains fairly similar for the nanowires with and without passivation: between one and 2 orders of magnitude. The latter indicates that the nonradiative recombination channels are still very active after applying the passivation films. This could either mean that the passivation is not very effective or that the surface is not the main cause for nonradiative losses in these nanowires. Considering that these passivation schemes have demonstrated very low surface recombination velocities on cubic germanium and cubic silicon, the former seems unlikely. Moreover, the quick screening of several other passivation schemes (Figure 5) shows that all tested films and pretreatments have only mild impact on the PL, which also hints at a small influence of the surface on the PL rather than a strongly limiting surface. These two reasons make it most likely that the surface is not one of the main nonradiative recombination channels in these nanowires. This statement is further reinforced by time-resolved photoluminescence measurements of SiGe nanowires with various diameters. In this experiment, no increase of the lifetime was found for an increasing diameter (Figure 6). This result also implies a surface that hardly influences the PL of the SiGe.

Since the lifetime of the nanowires seems not surface-limited, the measured lifetimes in Figure 6 represent merely a lower limit for the surface lifetime (τ_s_). From the latter, an upper limit for the surface recombination velocity of the native hex-SiGe surface can be estimated (*S*_eff,max_). Using the data of the nanowire with the smallest diameter, this results in
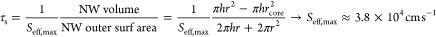
where *h* represents the length of the nanowire, *r* is the radius, and *r*_core_ is the radius of the GaAs core. This upper limit indicates a surface recombination velocity of the native hex-SiGe surface that is relatively low compared to typical surface recombination velocities of for example GaAs (*S*_eff_ ≈ 4 × 10^4^–10^7^ cm s^–1^)^25−28^ and InGaAs (*S*_eff_ ≥ 1.5 × 10^4^ cm s^–1^).^42^ Compared to InP (*S*_eff_ ≈ 10^2^–10^4^ cm s^–1^),^20−24^ the surface recombination velocity of hex-SiGe can be relatively large, although no definite conclusions can be drawn since our estimation for hex-SiGe concerns an upper limit.

Despite the tolerance of hex-SiGe alloys for relatively high surface recombination rates, it needs to be emphasized that metals or unannealed plasma-processed thin films (see Section 7 in the Supporting Information) may facilitate very high recombination rates, which can largely surpass the calculated upper limit for the surface recombination rate and still quench the PL. Also, improvements of the bulk quality may cause the surface to eventually become the limiting factor for the PL. Lastly, it is observed that the PL response of hex-SiGe nanowires can degrade over time when exposed to air (Section 8 in the Supporting Information). Conformal passivation films as presented in this work can be useful to prevent this, which we will investigate in detail in future work. With the surface unlikely to be the main cause for nonradiative surface recombination, several other possible loss mechanisms remain. To pinpoint the most likely candidate for this, it is useful to mention that Auger recombination is thought to be negligible.^11^ Likewise, recombination in the GaAs due to carriers moving from the hex-SiGe into the wurtzite GaAs core is improbably due to a band offset, which is believed to be type I (see Section 6 in the Supporting Information). With these candidates ruled out, the presence of substantial losses through recombination centers located in the bulk of the (Si)Ge material remains most likely. Additionally, nonradiative recombination centers can possibly be located at the interface of the wurtzite GaAs core and the hex-SiGe shell. The nonradiative recombination channel related to the lowest activation energy (*E*_a,1_ ≈ 2–8 meV) has a very low quenching rate (*C*_1_ ≈ 1–4 au) and may be tentatively attributed to the ionization of acceptor states involved in the PL of the hex-SiGe.^35−37^ Higher activation energies are most likely related to deep-level traps, i.e., effective recombination centers, which can consequently be attributed to the aforementioned defects in the bulk of the hex-SiGe material or those at the GaAs/SiGe interface. Regarding the atomistic nature of point defects in hex-SiGe, the recent work of Sun et al.^43^ is valuable. For *hex-Si*, they showed that single or double Si vacancies give rise to defects in the center region of the bandgap. A similar effect was observed for some types of interstitials (H and XT interstitial). This means that these types of defects are likely to be efficient recombination centers and hence could explain nonradiative bulk recombination in hex-Si. Although not shown in their work and hence not proven yet, one can imagine that for hex-SiGe similar defects may exist. Several other defects that are discussed in their work give only rise to shallow traps or states outside the bandgap, just like the recently discovered I3 basal stacking fault.^41^ These defects are therefore unlikely to cause strong nonradiative bulk recombination in hex-SiGe.

## 5 Conclusions

Several ultrathin (<25 nm) passivation layers that have demonstrated low surface recombination velocities on cubic germanium and silicon have been applied to hex-Ge and hex-SiGe nanowires. At room temperature and relatively high excitation densities (1–8 kW cm^–2^), these passivation schemes lead to only relatively small changes in the integrated photoluminescence compared to the nanowires with a native surface. Additionally, the lifetime of the nanowires with a native surface was not found to decrease with decreasing diameter. Considering these observations, we conclude that the hex-(Si)Ge surface is most likely not strongly influencing, hence not limiting, the PL in the high excitation regime. An upper limit for the surface recombination of *S*_eff,max_ < 3.8 × 10^4^ cm s^–1^ was estimated. The strong decrease in internal quantum efficiency with increasing temperature may consequently stem predominantly from nonradiative recombination in the bulk of the hex-(Si)Ge. The results of this research imply that for hex-SiGe-based devices with micrometer dimensions operating at relatively high excitation densities, like nanolasers, surface recombination will most likely not be a bottleneck. Despite not being strongly limiting, direct contact between the SiGe surface and layers that facilitate (very) high carrier recombination rates (e.g., metals, unannealed plasma-processed dielectrics, etc.) should still be avoided. The result of this research is considered useful for the realization of a hex-SiGe-based laser, which is an important milestone on the roadmap of silicon photonics.

## Supporting Information Available

Energy-dispersive X-ray spectroscopy (EDX) of SiGe nanowires coated with ALD Al_2_O_3_; parameters Arrhenius fits; photoluminescence spectra and excitation dependence of PL; details about the nanowires and the different passivation schemes used in Figure 5; time-resolved photoluminescence data; estimation of the band offset between hex-SiGe and wurtzite GaAs; deterioration of the PL after deposition of plasma-enhanced ALD Al_2_O_3_; and degradation of PL over time (PDF)
