# Supplementary material for: Low Surface Recombination in Hexagonal SiGe Alloy Nanowires: Implications for SiGe-Based Nanolasers
Source: ACS Appl Nano Mater. 2024 Jan 12;7(2):2343–51. doi: 10.1021/acsanm.3c05770 (PMC10825821; doi:10.1021/acsanm.3c05770)
Supplement: Supplementary file 1 — an3c05770_si_001.pdf [file an3c05770_si_001.pdf]

Supporting Information for

Low Surface Recombination in Hexagonal SiGe Alloy Nanowires: Implications for SiGe-based Nanolasers

Wilhelmus J. H. (Willem-Jan) Berghuis[1], Marvin A. J, van Tilburg[1], Wouter H. J. Peeters[1], Victor T. van Lange[1], Riccardo Farina[1], Elham M. T. Fadaly[1], Elsa C. M. Renirie[1], Roel J. Theeuwes[1], Marcel. A. Verheijen[1,2], Bart Macco[1], Erik P. A. M. Bakkers[1], Jos E. M. Haverkort[1,a)], Wilhelmus M. M. (Erwin) Kessels[1,b)]

[1]Eindhoven University of Technology, Postbus 513, 5600 MB Eindhoven, The Netherlands
[2]Eurofins Materials Science BV, High Tech Campus 11, 5656 AE Eindhoven, The Netherlands

[a)] Electronic mail: j.e.m.haverkort@tue.nl (corresponding address 1)
[b)] Electronic mail: w.m.m.kessels@tue.nl (corresponding address 2)

1) Energy Dispersive X-ray Spectroscopy (EDX) of SiGe nanowires coated with ALD Al_2O_3

With the help of a JEOL ARM 200F Transmission Electron Microscope (TEM) operated at 200 kV and equipped with a 100 mm^2 Centurio SDD energy dispersive x-ray (EDX) spectroscopy detector, GaAs/SiGe core/shell nanowires were examined after the deposition of ALD Al_2O_3 to see whether deposition of an oxide such as Al_2O_3 leads to clustering or segregation of Si or Ge. In Fig. S1, the results of this study are presented for a SiGe nanowire with composition $Si_{0.2}Ge_{0.8}$, diameter 0.06±0.005 µm, and length 1.7± 0.1 µm. The nanowire has been passivated with a 22 nm Al_2O_3 film. EDX was used to map the elements within the GaAs/SiGe core/shell nanowire. In Fig. S1a, a High-Angle Annular Dark-Field Scanning Transmission Electron Microscopy (HAADF-STEM) image of the investigated nanowires is shown. In Fig. S1b-f, the distributions of Ge, Si, Ga, As, and Al are displayed respectively. Most important observation is that from Fig. S1b and Fig. S1c, no clear signs appear for clustering or segregation of Si or Ge after deposition of the passivation layer.

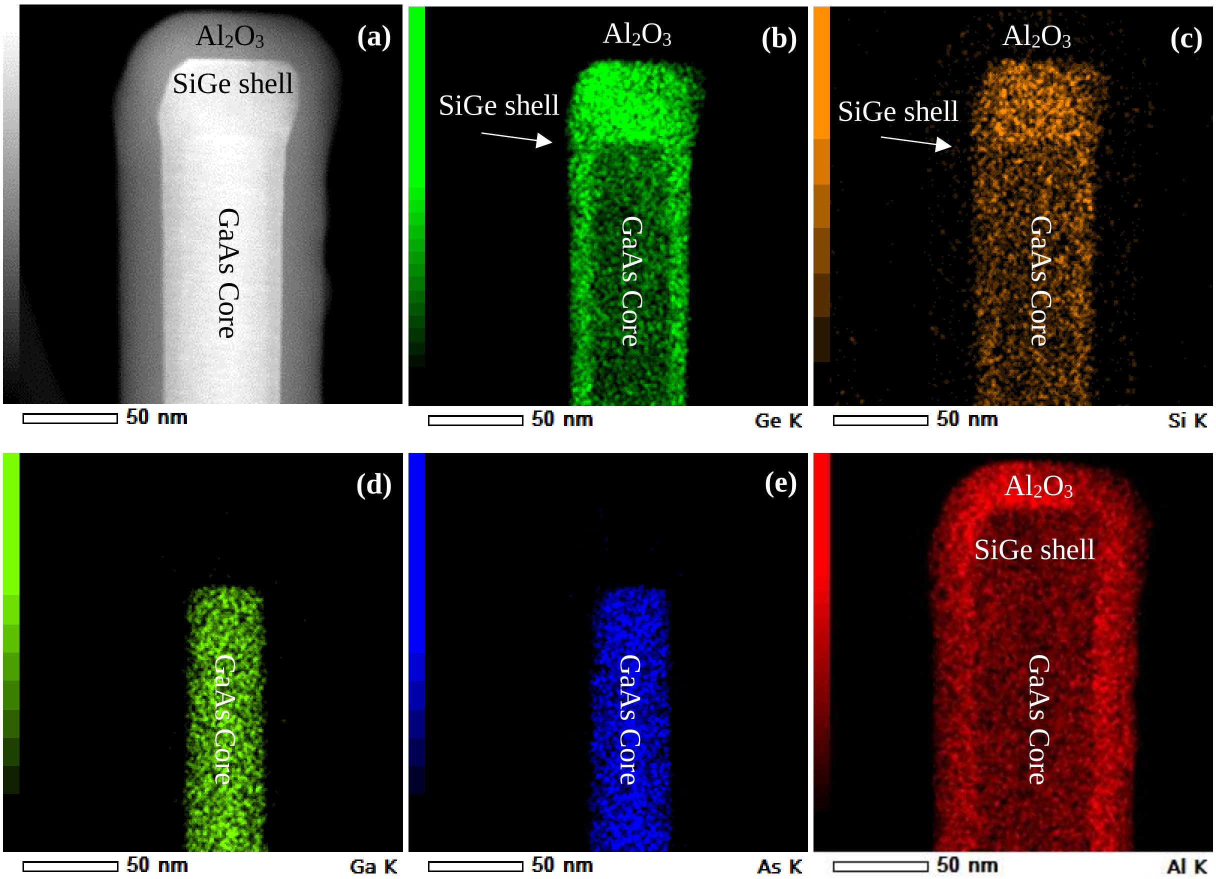

Fig. S1 (a) High-Angle Annular Dark-Field Scanning Transmission Electron Microscopy (HAADF-STEM) image of a GaAs/SiGe core/shell nanowire ($Si_{0.2}Ge_{0.8}$, length ≈ 1.7 µm, diameter ≈ 60 nm). Panels (b) to (f) show the distributions of the elements Ge, Si, Ga, As, and Al within the nanowire displayed in (a).

2) Parameters Arrhenius fits

Table S1 Overview of Arrhenius activation energies and quenching coefficients associated with the fits in Fig. 2 of the main text.

	As-grown	Passivated by Al_2O_3
$E_{a,1}$ (meV)	3.8 ± 0.3	3.2 ± 0.2
$E_{a,2}$ (meV)	25 ± 2	27 ± 2
C_1 (a.u.)	2.4 ± 0.4	2.6 ± 0.2
C_2 (a.u.)	183 ± 5	100 ± 2

Table S2 Overview of Arrhenius activation energies and quenching coefficients associated with the fits in Fig. 3 of the main text.

	As-grown	Passivated by a-Si:H/Al_2O_3
$E_{a,1}$ (meV)	2.69 ± 0.02	2.58 ± 0.7
$E_{a,2}$ (meV)	22 ± 3	21 ± 1
C_1 (a.u.)	4.3 ± 0.3	3.1 ± 0.1
C_2 (a.u.)	$(3 \pm 1) \times 10^1$	20 ± 2

Table S3 Overview of Arrhenius activation energies and quenching coefficients associated with the fits in Fig. 4 of the main text.

	As-grown	Passivated by PO_x/Al_2O_3
$E_{a,1}$ (meV)	1.6 ± 0.1	2.95 ± 0.05
$E_{a,2}$ (meV)	14.1 ± 0.9	19.7 ± 0.05
$E_{a,3}$ (meV)	$(7 \pm 2) \times 10^1$	$(9 \pm 1) \times 10^1$
C_1 (a.u.)	0.64 ± 0.05	2.38 ± 0.06
C_2 (a.u.)	13 ± 2	76 ± 7
C_3 (a.u.)	$(6 \pm 7) \times 10^{2}$ *	$(3 \pm 2) \times 10^4$

* The reliability of this value is questionable due to the limited temperature range of the data.

Table S4 Overview of Arrhenius activation energies and quenching coefficients associated with the fits in Fig. S5 of the supporting information.

	As-grown	Passivated by a-Si:H/Al_2O_3
$E_{a,1}$ (meV)	8.3 ± 0.4	4.1 ± 0.3
$E_{a,2}$ (meV)	42 ± 2	30 ± 2
C_1 (a.u.)	2.2 ± 0.2	1.1 ± 0.1
C_2 (a.u.)	60 ± 8	28 ± 3

3) Photoluminescence spectra and excitation dependence of PL

3.1) Al_2O_3 passivation

In Fig. S2a, the PL spectra are shown for hex-Si_0Ge_1 nanowires with and without a 22 nm plasma-enhanced ALD Al_2O_3 passivation film. The PL intensity is normalized with respect to the peak height of the as-grown sample. The shape of the spectra are very similar and the peak is positioned around 0.31 eV, which is expected for pure hex-Ge at ~300 K and a relatively high excitation density[1].

In Fig. S2b, the excitation dependence of the integrated PL is shown for hex-Ge nanowires with and without the Al_2O_3 passivation. The slopes derived from linear fits of the data in Fig. S1b yield values of about ≈ 1.12 (a.u.) and ≈ 1.13 (a.u.) for the as-grown wires and the passivated wires, respectively. The similar slope indicates no clear effect of passivation. The slope values lie between the value expected for pure radiative recombination (slope = 1) [1,2] and pure non-radiative recombination (slope = 2) [1,2], indicating a slight contribution of non-radiative recombination channels at this temperature, potentially arising from defects in the bulk of the nanowires as discussed in the main text. A more detailed discussion about the PL intensity as a function of excitation power density for hex-SiGe can be found in the work of Fadaly *et al.*[1] who base their discussion on earlier work on III/V semiconductors[2,3].

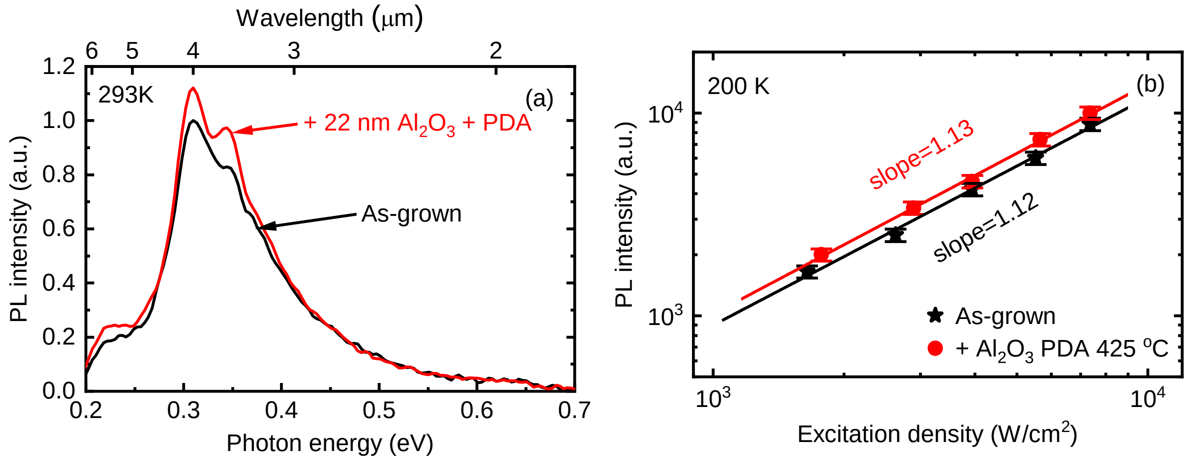

Fig. S2 (a) The photoluminescence spectra of hex-Ge nanowires without and with Al_2O_3 passivation stack measured at 293 K. (b) The integrated photoluminescence of hex-Ge nanowires as a function of excitation density at 200 K. The temperature of 200 K was chosen since it is an elevated temperature at which non-radiative recombination is active, yet this temperature is sufficiently low to still measure a PL signal at low excitation densities, hence allowing for a decent range of excitation densities.

3.2) a-Si:H/Al_2O_3 passivation

In Fig. S3, the PL spectra are shown for hex-$Si_{0.23}Ge_{0.77}$ nanowires with and without an a-Si:H/Al_2O_3 stack at a low and relatively high temperature: 4 K and 100 K. The PL intensity is normalized with respect to the peak height of the as-grown sample at 4 K. The peak is for both the as-grown and passivated wires located around 0.62 eV, which is approximately what one expected for hex-SiGe with this composition ($Si_{0.23}Ge_{0.77}$) [1].

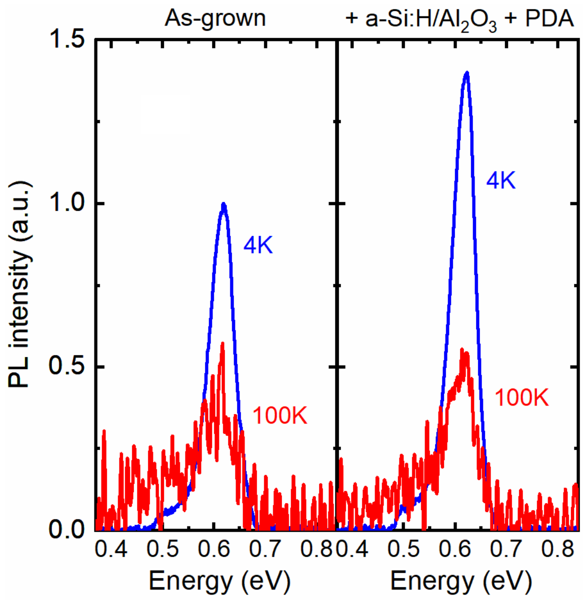

Fig. S3 The photoluminescence spectra of hex-$Si_{0.23}Ge_{0.77}$ nanowires without and with a-Si:H/Al_2O_3 passivation stack measured at 4 K (blue) and 100 K (red).

In Fig. S4a, the PL spectra are shown for hex-Ge nanowires with and without an a-Si:H/Al_2O_3 stack. The PL intensity is normalized with respect to the peak height of the as-grown sample. The shape of the spectra are virtually the same and the peak around 0.33 eV is approximately what is expected for pure hex-Ge at ~300 K and a relatively high excitation density[1].

In Fig. S4b, the excitation dependence of the integrated PL is shown for hex-Ge nanowires with and without the a-Si:H/Al_2O_3 passivation. The slopes derived from linear fits of the data in Fig. S1b yield values of about ≈ 1.2 (a.u.) and ≈ 1.47 (a.u.) for the as-grown wires and the passivated wires, respectively. These slopes are between the slope expected for pure radiative recombination (slope = 1)[2] and pure non-radiative recombination (slope = 2)[2], indicating a moderate contribution of non-radiative recombination at this temperature and excitation densities. The passivated sample yields a slightly

higher slope which is indicative for slightly more non-radiative recombination, while the temperature dependence of the PL (main text) and absolute PL intensity (main text) of this sample seem to indicate a mild improvement by the passivation. These improvements and degradations of the PL after applying the passivation stack are however relatively small and indicate mainly a limited influence of the surface (see the discussion section of the main text).

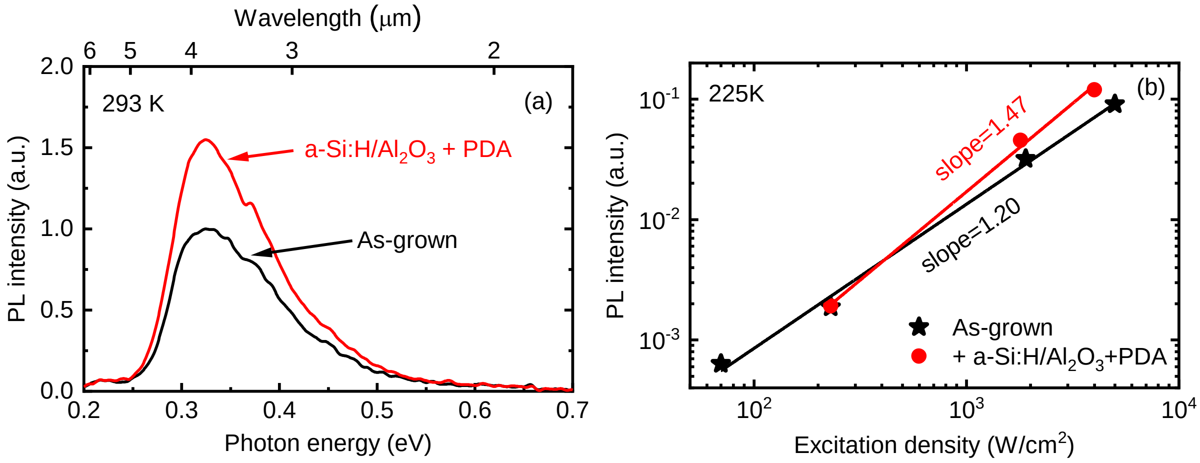

Fig. S4 (a) The photoluminescence spectra of hex-Ge nanowires without and with a-Si:H/Al_2O_3 passivation stack measured at 293 K. (b) The integrated photoluminescence of hex-Ge nanowires as a function of excitation density at 225 K. The temperature of 225 K was chosen since it is an elevated temperature at which non-radiative recombination is active, yet this temperature is sufficiently low to still measure a PL signal at low excitation densities, hence allowing for a decent range of excitation densities.

The effect on the integrated PL of the a-Si:H/Al_2O_3 stacks is shown in Fig. S5. The integrated PL intensities of the as-grown hex-Ge wires (black) and the passivated wires (red) have been normalized at 6 K. The PL quenching with temperature in Fig. S5 seems to be characterized by two knees for both the passivated and unpassivated wires. The Arrhenius fits yield activation energies of $E_{a,1} \approx 4.1 - 8.3$ meV and $E_{a,2} \approx 30 - 42$ meV for these two knees. When comparing the passivated and as-grown wires, it becomes evident that they show very similar behavior. The PL intensity of the passivated wires decrease a little bit less with increasing temperature ($C_{2,pass} \approx 28$ a.u. vs $C_{2,unpass} \approx 60$ a.u.) and the absolute PL intensity is about 30% higher at room temperature. The latter hints at a slight reduction in non-radiative recombination pathways, presumably related to the surface.

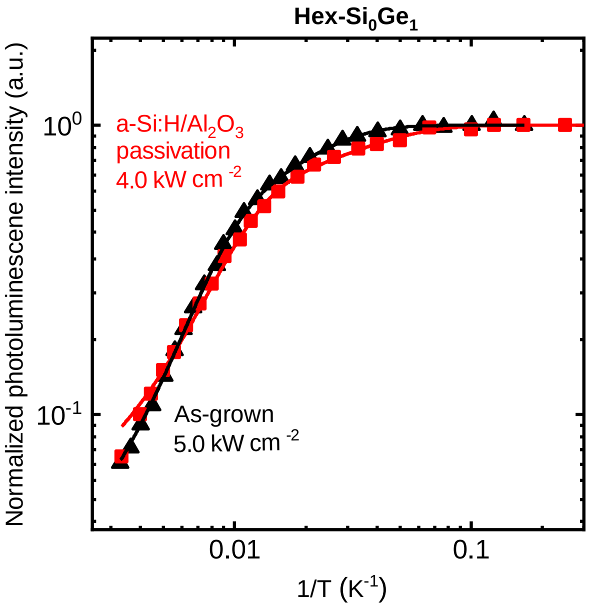

Fig. S5 Arrhenius representation of the photoluminescence intensity as a function of inverse temperature for hex-Si_0Ge_1 nanowires with and without a-Si:H/Al_2O_3 passivation stack. The integrated PL intensities are normalized to their respective intensity at 6 K. The data is fitted with the Arrhenius equation (solid lines through the data, parameters listed in Section 2 of the SI).

3.3) PO_x/Al_2O_3 passivation

In Fig. S6a, the PL spectra for hex-$Si_{0.23}Ge_{0.77}$ nanowires without and with a PO_x/Al_2O_3 stack are shown. The PL intensity is normalized with respect to the peak height of the as-grown sample at 4 K. The peak for the passivated nanowires is positioned around a slightly lower photon energy (0.56 eV) than the as-grown wires (0.60 eV). As discussed in the main text, the PL of the wires with PO_x/Al_2O_3 passivation show still a strong quenching with temperature of about two orders of magnitude between 4 K and 300 K. The nanowires with the PO_x/Al_2O_3 stack decrease also substantially faster with increasing temperature compared to the as-grown nanowires. Additionally, the absolute integrated PL at 160 K (the highest measurable temperature) is 40% lower after applying the passivation film. These observations indicate no reduction in surface recombination by the PO_x/Al_2O_3 stack.

Yet, there are some not fully understood observations: for low temperatures, the PL of the passivated wires is substantially higher than for the as-grown wires (Fig. S6a and Fig. S6b). Although sample inhomogeneity might explain a factor 2-3 difference in PL intensity, this is remarkable since at these temperatures, especially at 4 K, non-radiative recombination is generally believed to be negligible. This general assumption is backed up by preliminary TRPL measurements at 4 K (Fig. S7b), which did not

reveal a higher lifetime for the passivated wires compared to the as-grown wires. Consequently, at these temperatures one does not expect any effect from the passivation stack. This large difference in absolute integrated PL at low temperatures is not understood and seems most likely to arise from an additional unknown effect. Apart from sample inhomogeneity, one can speculate that the positive fixed charge density in the PO_x/Al_2O_3 [4] induces a beneficial electric field in these *n*-type wires, which can alter the PL. Further investigation of this phenomenon is required, but falls outside the scope of this research.

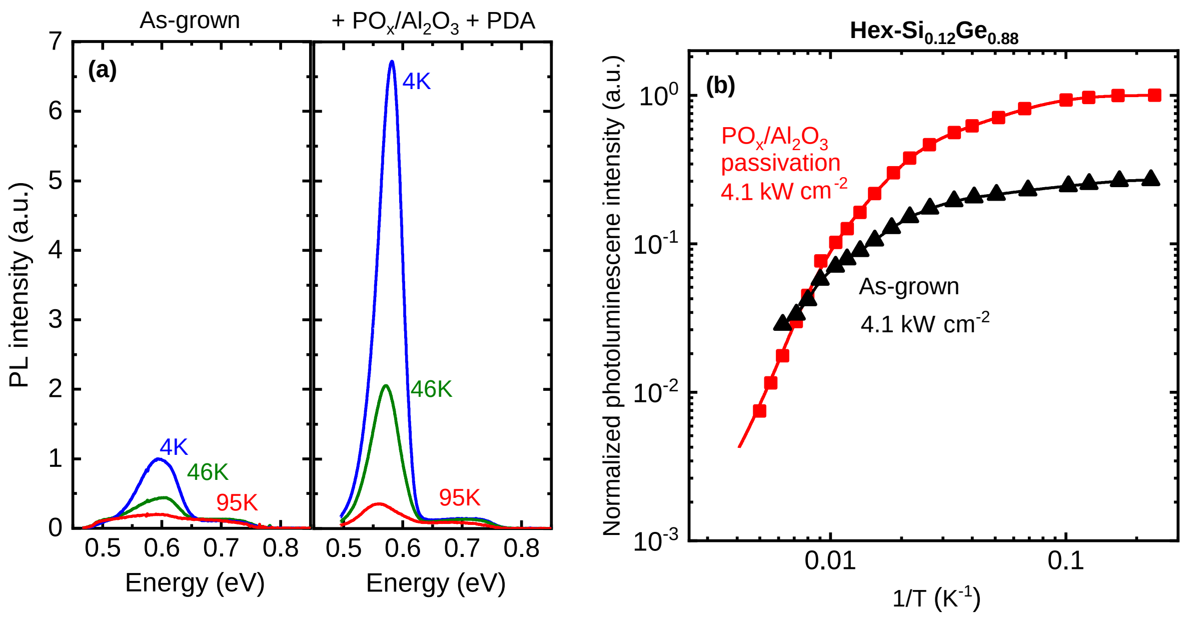

Fig. S6 (a) The photoluminescence spectra of hex-$Si_{0.23}Ge_{0.77}$ nanowires without and with PO_x/Al_2O_3 passivation stack measured at 4 K (blue), 46 K (green), and 95 K (red). (b) The integrated PL of hex-$Si_{0.12}Ge_{0.88}$ nanowires with and without PO_x/Al_2O_3 passivation as a function of inverse temperature. The integrated PL is normalized with respect to the PL intensity of the passivated wires at 4 K.

Regarding the supplementary TRPL measurements displayed in Fig. S7, the following can be said. The measurements were performed on nanowire ensembles using the TRPL setup described in the Experimental Section of the main text. The laser spot probes approximately 3-4 nanowires of the ensembles simultaneously. The TRPL data (Fig. S8) could be fitted with a biexponential model, i.e., two different lifetimes can be distinguished. The two lifetimes for the as-grown and passivated wires are displayed in Fig. S7 for both 4 K and 300 K. The initial lifetime of the decay is reported in Fig. S7a and the tail lifetime of the decay is reported in Fig. S7b. Note that the lifetimes at 4 K might also be reasonably well fitted using a mono-exponential model (Fig.S8a-b). The tail lifetime at 4 K, which dominates the decay, is very similar for the passivated and as-grown wires (Fig. S7b). At 300 K, the

biexponential model is essential to describe the data (Fig. S8c-d). The initial lifetime is very similar between the as-grown and passivated wires, but the tail lifetime shows a difference. The difference is approximately a factor two. This difference is in contrast with the integrated PL at elevated temperatures (Fig. S6b). The latter showed a higher PL for the as-grown wires. This contradiction is not completely understood yet and may also be related to the positive fixed charge in the passivation film. Additionally, a relatively stronger contribution of the substrate in this experiment could play a role. The newer generation of nanowire ensembles features namely a larger pitch, i.e., there is relatively more substrate area not covered by nanowires. Despite using a filter to remove the substrate contribution, the tail of the substrate's PL peak may still contribute to the spectrum (Fig. S6a) when the substrate PL peak is relatively strong compared to the nanowire PL peak. The latter could complicate the interpretation of the Arrhenius experiment, possibly leading to some error and hence discrepancy between the lifetime and integrated PL. This is still a topic of debate. Most important is that the differences in both the PL intensities and the lifetimes are maximal a factor two, which cannot explain the large decrease in PL emission when the wires are heated from 4 K to 300 K. Neither it could explain the large difference in lifetime between 4 K and 300 K and hence the surface seems unlikely the main reason of this.

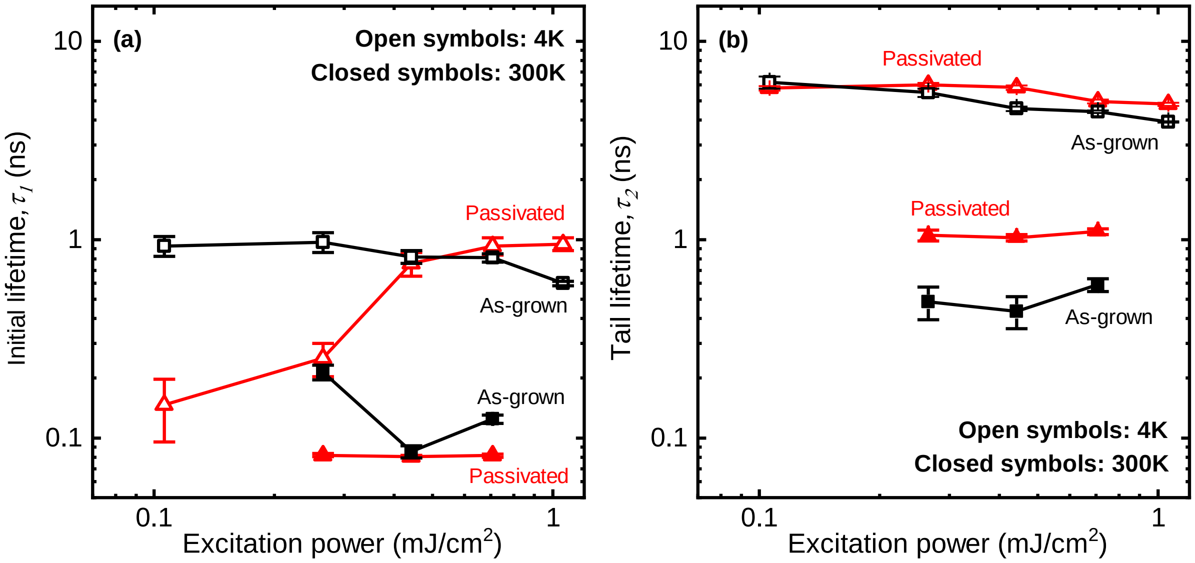

Fig. S7 Average lifetimes of $Si_{0.23}Ge_{0.77}$ nanowires as a function of excitation density at 4 K and 300 K. In (a) the initial lifetime of the TRPL decay is displayed and in (b) the tail lifetime of the decay. Typical raw data can be found in Fig. S8. The lifetime of wires with PO_x/Al_2O_3 passivation (red data) are shown together with the lifetimes of wires without passivation (black data). For each datapoint about 3-4 nanowires were averaged. The samples for this experiment concern H06950 and H06950-2.

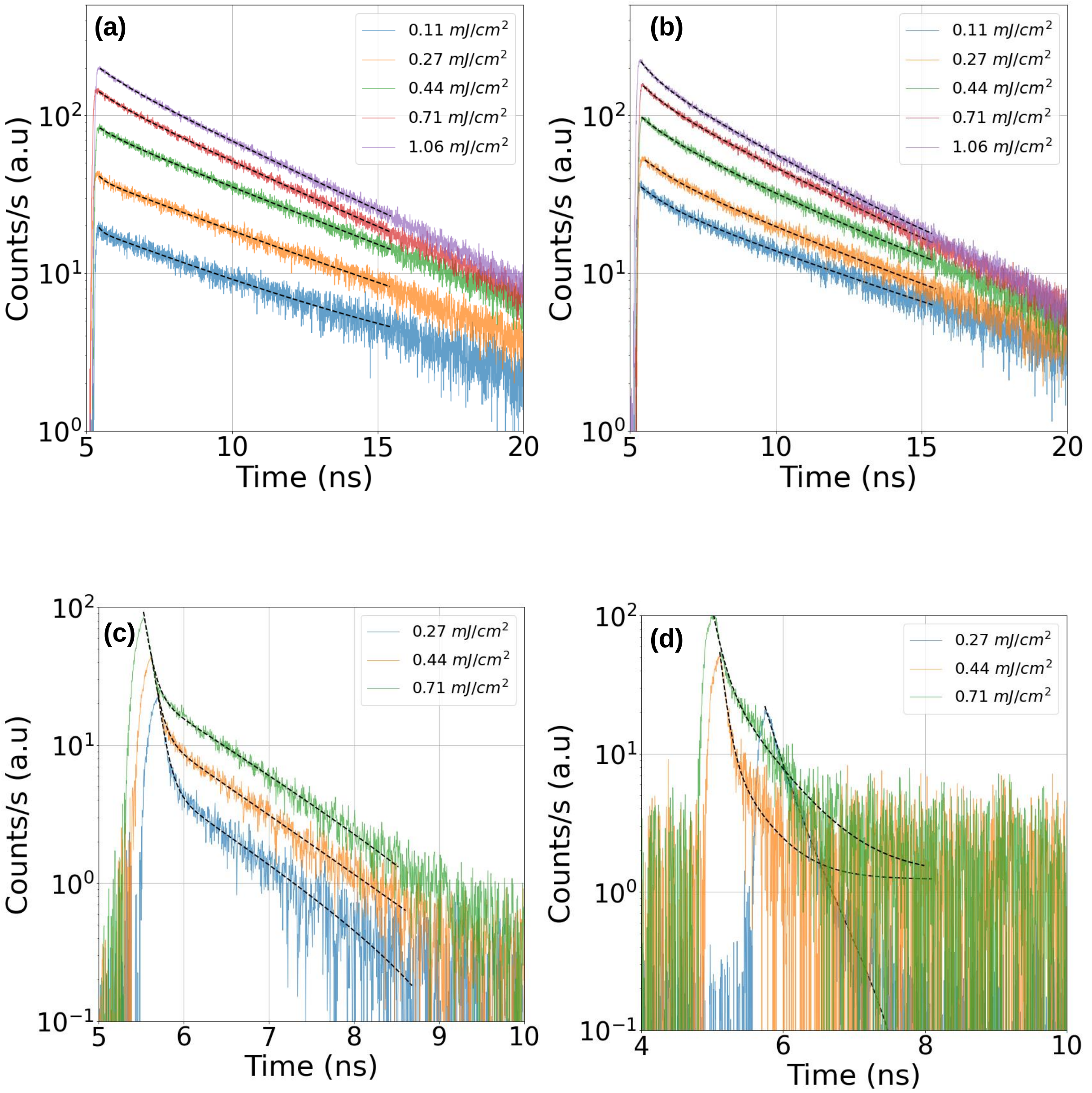

Fig. S8 Typical time resolved photoluminescence measurements obtained from nanowire ensembles used for the Arrhenius plot shown in Fig. 4 of the main text. The data was fitted with a biexponential model of which the two lifetimes are presented in Fig. S8. The TRPL decay is shown for passivated wires at 4 K (a), as-grown wires at 4 K (b), passivated wires at 300 K (c), and as-grown wires at 300 K (d).

4) Details about the nanowires and the different passivation schemes used in Fig. 5

Table S5 Overview of nanowire samples and measurement conditions of Fig. 5 of the main text.

Sample name	Nanowire composition	Diameter (μm)	Length (μm)	Passivation scheme	Surface treatment	T_{PDA} (°C)	T_{PL} (K)	Excitation density ($kWcm^{-2}$)
H06235	Si_0Ge_1	0.7±0.1	6.5±0.5	Al_2O_3 (22 nm)	1% $HF_{(aq)}$	425	293	7.4
H06235	Si_0Ge_1	0.7±0.1	6.5±0.5	GeO_2 (2.7 nm)	O_2 plasma	425	293	7.4
H06235	Si_0Ge_1	0.7±0.1	6.5±0.5	a-Si:H (80 nm)	20% $HBr_{(aq)}$	250	293	7.4
H06062	Si_0Ge_1	0.5±0.1	1.8±0.3	Al_2O_3 (20 nm)	none	425	293	5.1
H05735	Si_0Ge_1	Unk.	Unk.	GeO_2/Al_2O_3 (-/22 nm)	UV-ozone	425	293	5.1.
H06801	Si_0Ge_1	1.7±0.6	2.9±0.5	a-Si:H/Al_2O_3 (2/11nm)	20% $HBr_{(aq)}$	325	293	4
H06062	Si_0Ge_1	0.5±0.1	1.8±0.3	PO_x/Al_2O_3 (5/11nm)	none	400	293	7.4
H06950-2	$Si_{0.23}Ge_{0.77}$	0.617±0.02	5.62±0.02	PO_x/Al_2O_3 (4/10nm)	none	250	160	4.1
H05916	$Si_{0.23}Ge_{0.77}$	0.43 ± 0.07	1.6 ± 0.4	Al_2O_3 (22 nm)	1% $HF_{(aq)}$	425	89	6.8
H07771	$Si_{0.23}Ge_{0.77}$	2.08 ± 0.03	8.3 ± 0.3	a-Si:H/Al_2O_3 (2/11nm)	20% $HBr_{(aq)}$	325	149	0.85

5) Time resolved photoluminescence data

For the TRPL measurements used to make Fig. 6 of the main text, individual SiGe wires were mechanically placed onto a gold coated silicon wafer (5-7 for each data point). A SiO_2 layer was deposited on top of the gold to prevent direct contact between the wires and the gold. The individual wires were excited with a femtosecond pulsed laser operating at a wavelength of 1030 nm with a pulse frequency of 40 MHz, pulse width of 100 fs, and spotsize of ~3 μm. A superconducting nanowire single-photon detector (SNSPD) from the brand Single Quantum was used to measure the PL signal as a function of time. The reflection from the laser was filtered out using a 1350 nm long pass filter. The lifetime is extracted from the time resolved PL measurements by fitting the PL decay as a function of time with a mono exponential decay ($y = y0+A*\exp(-x/\tau)$). Figure S9a− i shows in each column typical TRPL data for nanowires with diameters of: ~0.6, ~1, and ~1.2 μm, respectively. The red lines through the data represent the exponential fit used to extract the lifetimes reported in Fig. 6 of the main text.

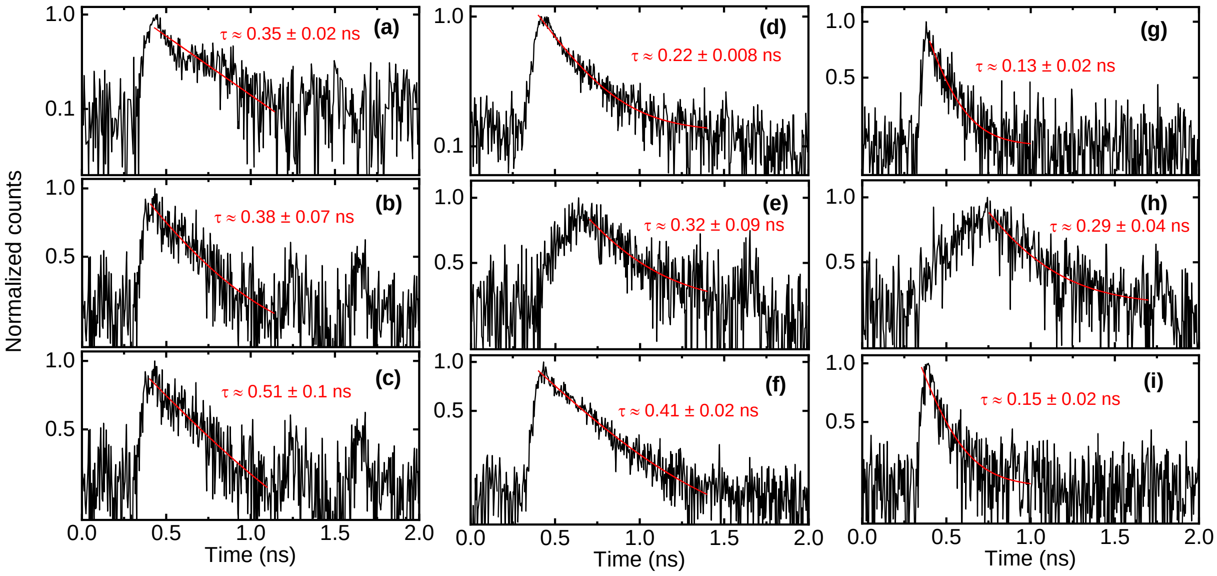

Fig. S9 Typical time resolved photoluminescence measurements obtained from individual nanowires originating from sample H6950 (a-c), H6948 (d-f), and H6988 (g-i) . The diameter of the nanowires on these samples is 0.617 ± 0.02 μm, 1.0 ± 0.01 μm , and 1.2 ± 0.01 μm. respectively (see also Table 1 of the main text). The data was acquired at the room temperature (300K). The red lines display the mono exponential fits (y = y0+A*exp(-x/τ)) .

6) Estimation of the band offset between hex-SiGe and wurtzite GaAs

By combining the work of Belabbes *et al.*[5], Van de Walle *et al.*[6], and Ahtapodov *et al.*[7], one can make a tentative estimation of the band offsets between wurtzite GaAs and hexagonal Si_xGe_{1-x} ($0<x<0.25$). In this estimation, the conduction band has an offset of approximately 0.5 eV for hex-Ge and wurtzite GaAs, which decreases to 0.3 eV for the conduction band offset between wurtzite GaAs and hexagonal $Si_{0.25}Ge_{0.75}$. The valence band offset is in either case about 0.6 eV. Not all references report uncertainties of their numerical results. Van de Walle *et al.*[6] reports an uncertainty of 0.2 eV in their band off sets, which thereby forms the lower limit for the accuracy of this estimation. From this result, it becomes clear that the band offset between the GaAs core and the hex-Si_xGe_{1-x} shell is type I, i.e., straddling gap. This means carriers are very unlikely to move from the hex-SiGe shell into the GaAs core. Details about the estimation can be found in Table S6 below.

Table S6 Conduction band offsets (CBO) and valence band offsets (VBO) between various semiconductors derived from (numerical) the work of Belabbes *et al.*[5], Van de Walle *et al.*[6], and Ahtapodov *et al.*[7].

	CBO (eV)	VBO (eV)	Reference
Wurtzite GaAs w.r.t. zincblende GaAs	0.120	0.120	[7]
Zincblende GaAs w.r.t. cubic Ge	0.104	-0.706	[6]
GaAs wurtzite w.r.t. cubic Ge	0.224	-0.586	[6,7]
Cubic Ge w.r.t. hex $Si_{0.25}Ge_{0.75}$	0.054	-0.004	[5]
Cubic Ge w.r.t. hex-Ge	0.252	-0.060	[5]
Wurtzite GaAs w.r.t. hex-$Si_{0.25}Ge_{0.75}$	0.278	-0.590	[5–7]
Wurtzite GaAs w.r.t. hex-Ge	0.476	-0.646	[5–7]

7) Deterioration of the PL after deposition of plasma-enhanced ALD Al_2O_3

Fig. S10 shows the effect of 22 nm plasma-enhanced ALD (PEALD) Al_2O_3 on hex-Ge before and after annealing. A clear deterioration of the PL occurs after applying the Al_2O_3 film. The PL is recovered after applying a low temperature post-deposition anneal (PDA). The deterioration in PL is likely due to a *very* defective interface between the hex-Ge and the Al_2O_3. This observation aligns with passivation studies on cub-Ge[8] and cub-Si[9] with PEALD Al_2O_3. These studies have demonstrated that the surface recombination velocities (S_{eff}) can be orders of magnitude higher before applying the post-deposition anneal to the Ge/Al_2O_3 or Si/Al_2O_3 interface and can yield very high SRV values. As in the specific case of Fig. S10, the interface between the hex-Ge and as-deposited Al_2O_3 seems substantially limiting the PL. The very high recombination rates facilitated by unannealed dielectrics such as PEALD Al_2O_3 appear to make the surface a limiting factor for the PL. A low temperature PDA is key in resolving this.

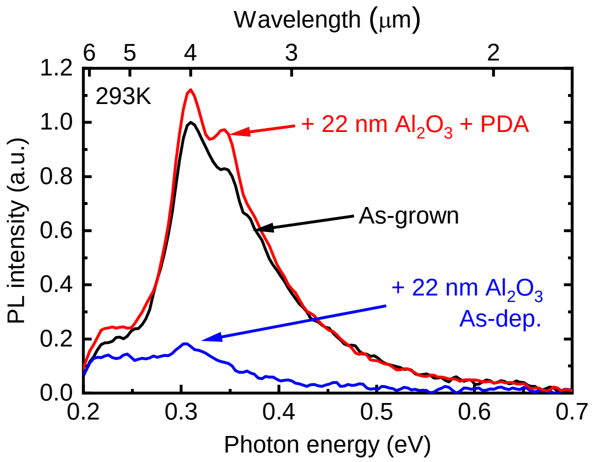

Fig. S10 The photoluminescence spectra of hex-Ge nanowires with and without Al_2O_3 passivation film measured at ~293 K using an excitation density of 7.4 kW cm^{-2}. The spectrum of the as-grown wires is depicted in black. The spectrum after the deposition of a 20 nm Al_2O_3 passivation layer is shown in blue. The red spectrum shows the PL after annealing the passivated nanowires at 425 ^{o}C. All spectra have been scaled with the maximum height of the as-grown spectrum.

8) Degradation of PL over time

Fig. S11 demonstrates that exposure of the hex-(Si)Ge nanowires without passivation layer to air can gradually lead to some degradation of the integrated PL. The mechanism behind this observation is still under investigation. Although not completely clear yet, one can speculate that it is related to a serious increase in surface recombination rate due to strong degradation of the native germanium oxide, which is known to be rather unstable (e.g. hygroscopic).[10–12]

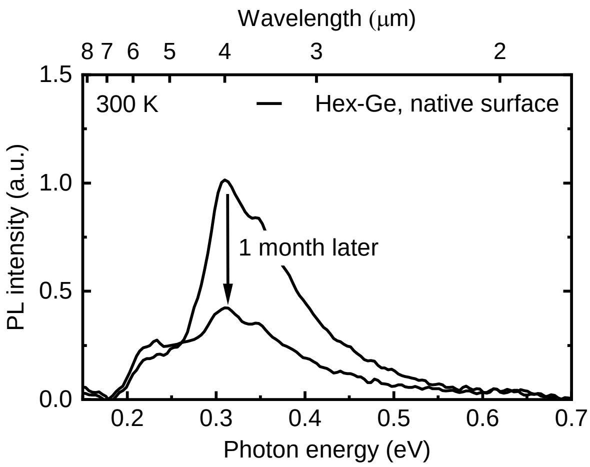

Fig. S11 The photoluminescence spectra of hex-Ge nanowires with native surface measured at ~293 K using an excitation density of 7.4 kW cm^{-2} right after taking it from the glovebox (upper spectrum) and after 1 month storage in ambient air, without any precautions (lower spectrum).

References

1. E. M. T. Fadaly, A. Dijkstra, J. R. Suckert, D. Ziss, M. A. J. Van Tilburg, C. Mao, Y. Ren, V. T. Van Lange, K. Korzun, S. Kölling, M. A. Verheijen, D. Busse, C. Rödl, J. Furthmüller, F. Bechstedt, J. Stangl, J. J. Finley, S. Botti, J. E. M. Haverkort, and E. P. A. M. Bakkers: Direct-bandgap emission from hexagonal Ge and SiGe alloys. *Nature* **580**, 205–209 (2020).

2. Y.-S. Yoo, T.-M. Roh, J.-H. Na, S. J. Son, and Y.-H. Cho: Simple analysis method for determining internal quantum efficiency and relative recombination ratios in light emitting diodes. *Appl. Opt.* **102**, 211107 (2013) (2013).

3. T. Schmidt, K. Lischka, and W. Zulehner: Excitation-power dependence of the near-band-edge photoluminescence of semiconductors. *Phys. Rev. B* **45**, 8989–8994 (1992).

4. R. J. Theeuwes, W. J. H. Berghuis, B. Macco, and W. M. M. Kessels: Excellent Passivation of Germanium Surfaces by PO_x/Al_2O_3. *Appl. Phys. Lett.* **123**, 091604 (2023).

5. A. Belabbes, S. Botti, and F. Bechstedt: Band lineup at hexagonal Si_x/Ge_{1-x}//SiyGe_{1-y}/ alloy interfaces. *Phys. Rev. B* **106**, 1–14 (2022).

6. C. G. Van De Walle: Universal alignment of hydrogen levels in semiconductors and insulators. *Phys. B Condens. Matter* **376–377**, 1–6 (2006).

7. L. Ahtapodov, H. Kauko, A. M. Munshi, B. O. Fimland, A. T. J. Van Helvoort, and H. Weman: Determination of GaAs zinc blende/wurtzite band offsets utilizing GaAs nanowires with an axial GaAsSb insert. *J. Appl. Phys.* **122** (2017).

8. W. J. H. Berghuis, J. Melskens, R. J. Theeuwes, B. Macco, M. A. Verheijen, and W. M. M. Kessels: Surface Passivation of Germanium by Atomic Layer Deposited Al_2O_3 Nanolayers. *J. Mater. Res.* **36**, 571–581 (2021).

9. G. Dingemans, R. Seguin, P. Engelhart, M. C. M. Van De Sanden, and W. M. M. Kessels: Silicon surface passivation by ultrathin Al_2O_3 films synthesized by thermal and plasma atomic layer deposition. *Phys. status solidi - Rapid Res. Lett.* **4**, 10–12 (2010).

10. Q. Xie, S. Deng, M. Schaekers, D. Lin, M. Caymax, A. Delabie, X. P. Qu, Y. L. Jiang, D. Deduytsche, and C. Detavernier: Germanium surface passivation and atomic layer deposition of high-k dielectrics - A tutorial review on Ge-based MOS capacitors. *Semicond. Sci. Technol.* **27**, 1,12 (2012).

11. M. Houssa and E. Chagarov: Surface Defects and Passivation of Ge and III – V Interfaces. No. May 2014 (2009).

12. T. Hanrath and B. A. Korgel: Chemical Surface Passivation of Ge Nanowires. *J. Am. Chem. Soc.* **126**, 15466–15472 (2004).
